# Diversity of *Leptospira* Species and Their Rodent Reservoirs in the Guinean Forest

**DOI:** 10.3390/microorganisms13040833

**Published:** 2025-04-07

**Authors:** Siba Pricemou, Barré Soropogui, Fanta Bérété, Michel Bossou Beavogui, Aboubacar Samoura, Mathieu Picardeau, Pascale Bourhy, Noël Tordo, Solène Grayo

**Affiliations:** 1 Biodiversity and One Health Group, Institut Pasteur de Guinée, Conakry BP 4416, Guinea; sibapricemo@gmail.com; 2 Centre de Recherche en Virologie, Laboratoire des Fièvres Hémorragiques Virales de Guinée (CRV-LFHVG), Conakry BP 4416, Guinea; barresoropogui@gmail.com (B.S.); fantaberete012015@gmail.com (F.B.); 3 Office Guinéen des Parcs Nationaux et Réserves de Faune, Ministère de l’Environnement et du Développement Durable (MEDD), Conakry BP 4416, Guinea; beamichel68@gmail.com (M.B.B.); aboubacarsamoura7@gmail.com (A.S.); 4 Biology of Spirochetes Unit, National Reference Center for Leptospirosis, Institut Pasteur, 75015 Paris, France; mathieu.picardeau@pasteur.fr (M.P.); pascale.bourhy@pasteur.fr (P.B.); 5 Institut Pasteur de Guinée, Conakry BP 4416, Guinea; noel.tordo@pasteur.fr

**Keywords:** *Leptospira*, reservoir, micromammal, Guinea, West Africa, bacterial zoonoses

## Abstract

Leptospirosis is a bacterial zoonosis caused by pathogenic species from the genus *Leptospira*. Infection mostly occurs through indirect contact with environmental water contaminated with the urine of reservoir animals. Information on the circulation of leptospirosis in West Africa, as well as its potential reservoir hosts, is limited. Therefore, we carried out trapping surveys in the Guinean forest in November 2022, and samples were collected from 42 micromammals. The animals were both morphologically and genetically identified. The lungs and kidneys were screened for *Leptospira* using *Lfb1*-gene-targeting real-time PCR, and positive samples were genotyped based on the polymorphic *Lfb1* gene. *Leptospira* species were detected in the kidneys of three micromammals: *Mastomys natalensis*, *Lophuromys sikapusi*, and *Rattus rattus*. *Leptospira borgpetersenii* was identified in *Rattus rattus* and *Mastomys natalensis* that were captured in two different villages. The phylogenetic analysis indicated that this subspecies had previously been detected in one patient in Mayotte, but the reservoir was not identified. A new subspecies of *Leptospira kirschneri* was isolated in *Lophuromys sikapusi* from the same village as the *Mastomys natalensis* positive for *L. borgpetersenii*. The high diversity of both the reservoirs and *Leptospira* species in the Guinean forest indicates that we should study other natural regions and reinforce communities’ awareness of *Leptospira* infection risks in Guinea.

## 1. Introduction

Leptospirosis is a bacterial zoonotic disease that the World Health Organization (WHO) considers a public health problem [1,2]. It affects more than one million people worldwide and is responsible for about 60,000 deaths per year [3]. Leptospirosis is caused by a large variety of pathogenic *Leptospira* spp., with *Leptospira interrogans*, *Leptospira borgpetersenii*, and *Leptospira kirschneri* being the most abundant species circulating in humans and animals [4]. Clinical manifestations of leptospirosis range from a mild febrile illness to life-threatening renal failure, pulmonary hemorrhage, and/or cardiac complications. The fatal form is known as Weil’s disease [5]. *Leptospira* spp. are maintained in the renal system in several wild, livestock, and domestic animals and can be excreted in urine for several months. Human leptospirosis is mainly transmitted through environmental sources such as water, vegetables, soil, or dust contaminated by the urine of infected hosts, mainly rodents [6,7].

*Leptospira* transmission is often associated with poor hygiene, inadequate waste disposal, and urban overcrowding in the developing world. Deforestation, increased livestock farming, and agriculture contribute to transmission in rural areas [8]. Among the animals produced for consumption, cattle and pigs are relatively susceptible to infection, resulting in production losses, including reduced milk yields, reproductive failure, and abortions [9,10]. In 2019, the estimated average annual cost of leptospirosis due to loss of productivity was USD 29.3 billion.

Leptospirosis mainly affects people living in resource-poor settings, especially in tropical and subtropical regions where humans and animals live in close contact [11]. Although it is known that leptospirosis circulates in both humans and animals in sub-Saharan Africa (SSA), the disease does not seem to be taken seriously, and the most recent data on leptospirosis seroprevalence come from a limited number of countries [4,12,13].

For example, studies in Nigeria showed a high prevalence of pathogenic *Leptospira* in wild animals (around 80%, mostly in antelope and greater cane rats), which was often associated with tubular nephrosis [14]. *L. interrogans* was detected mainly in the kidneys and urine of rodents (*Rattus norvegicus* and *Cricetomys gambianus*) and cattle, with occurrences of about 70% and 30%, respectively [15,16]. A study conducted in the same area showed that males were four times more likely to be affected by leptospirosis, and the source of the animals’ water was five times more likely to be contaminated with *Leptospira* spp. [17].

In Benin, due to the emergence of leptospirosis in a bordering country and huge landscape changes (urbanization and maritime trade intensification), several studies have been carried out in the capital, Cotonou, to characterize the main reservoir, identify risk areas, and estimate the impact of the season on leptospirosis exposure [18,19,20,21]. Between 2009 and 2017, the results showed an average leptospirosis prevalence of 12.9%, and rodent-borne leptospirosis exposure varied widely depending on the area [21]. The native *Mastomys natalensis* was mostly infected by *L. borgpetersenii*, whereas the invasive *R. novergicus* was infected by *L. interrogans*, suggesting a possible host specificity. Curiously, *L. kirschneri* has only been recovered from African shrews (*Crocidura* spp.), insectivores that usually inhabit wild areas [21]. These three *Leptospira* spp. were also found in the kidneys of slaughtered cattle from an abattoir as well as the poor area of Cotonou, with an 18% seroprevalence [18]. Between 2016 and 2018, a long-term survey in Cotonou confirmed that the seroprevalence of leptospirosis within Cotonou varied according to the area and period of collection, but the season did not appear to affect the trend [19]. The flood season did not seem to be a risk factor, but standing water, such as wastewater, could be a source of rodent infection [19,21]. This survey also brought to light the great diversity of *Leptospira* species and the coexistence of different species at the quarter and house levels [19,22]. The diversity and abundance of reservoir hosts within Cotonou, mostly rodent species, complicates our understanding of the ecology of *Leptospira* and the ability to implement preventive measures.

The SSA region, particularly the coastal cities, is facing massive urbanization and the intensification of maritime trade, increasing human–animal interactions. Due to their large number and proximity to humans and domestic animals, rats (*Rattus* spp.) play a major role in the epidemiology of leptospirosis. With the exception of Togo (due to a lack of research), leptospirosis has been reported in rats and other endemic rodent species along the SSA coast [20] and in urban and rural areas of Nigeria [16], Benin [21], Ghana [23], the Ivory Coast [24], and even Senegal [25].

Despite the geographical location of Guinea, only one study has been carried out. It was conducted in the capital, Conakry, in 2004 [26]. There is a lack of knowledge about the reservoir animals of potentially pathogenic *Leptospira* species and limited epidemiological data on the disease. However, Guinea is confronted with the endemic circulation of Lassa fever (LF), a rodent-borne disease caused by Lassa virus (LASV), which is carried by *Mastomys* spp. [27,28,29]. Several surveys carried out in different regions of Guinea show a higher frequency of two species, *Mastomys* spp. (an endemic species) and *Rattus* spp. (an invasive species), which are mostly found inside houses. These species have already been shown to transmit some zoonoses to humans in the domestic spaces of rural areas [30].

The Guinean forest in the southeastern part of the country has high ecosystem richness (in terms of fauna and flora) and extensive pastoral areas (crops, pigsties, and ruminant farms). It hosts a great diversity of micromammal species, including rodents, shrews, and bats. The first hantavirus found on the continent, Sangassou virus (SANGV), was discovered here in African wood mice (*Hylomyscus simus*) in 2006 [31], and a second one, Tanganya virus (TGNV), was found in Therese’s shrews (*Crocidura theresae*) in 2007 [32].

Unfortunately, anthropogenic activities (deforestation and mining exploration) in the Guinean forest are bringing humans into closer contact with wildlife, which have previously been identified as a hotspot for emerging infectious diseases, such as Ebola virus disease [33]. The present work aimed to investigate the occurrence of *Leptospira* spp. in different species of micromammals captured in villages (at peridomestic and domestic sites) in the protected natural area of the Guinean forest.

## 2. Materials and Methods

### 2.1. Capture of Micromammals

Rodent trapping took place in November 2023 at the end of the rainy season in 3 villages, Sangassou (8.615504; −9.475624), Balassou (8.400137; −9.312699), and Seredou (8.400137; −9.312701), in the prefecture of N’Zérékoré (southeast region). The first site, Sangassou, is an isolated village in a grassland and savannah forest where the first hantavirus in Africa was discovered in 2006 in *H. simus* [31]. The two remaining sites are humid environments near a protected rainforest.

Sherman live traps (2 or 3 traps per area) were set in domestic areas (houses, granaries, and crop stores), backwater areas, and/or near agricultural areas growing rice, peanuts, etc., with the support of local guides. A mixture of peanuts, flour, and fish was used as bait. The traps were monitored early in the morning for 3 consecutive days. Cages containing captured animals were promptly transferred to a bag at the inspection site. These cages were cleaned prior to being reinstalled. The captured micromammals were phenotypically classified in the field using key morphological characteristics such as body measurements (head and body, tail, foot, and ear lengths) and fur color. Lateral, ventral, and dorsal pictures were taken, and the sex of each animal was recorded.

### 2.2. Sample Collection

For this study, all animals were trapped in houses or peridomestic areas, considering them as “invasive” animals. Non-invasive (tail and ears) and invasive samples (kidney and lung) were taken from all 42 micromammals using the following process. The micromammals were anesthetized with halothane and subsequently euthanized via cervical dislocation. Following the disinfection of the abdomen with alcohol, the rodents were biopsied. The tail, kidneys, and lungs were collected and stored at −20 °C in an RNA/DNA shield in a portable ice box that was connected to the vehicle power supply to maintain the temperature. The samples were then sent to Institut Pasteur of Guinea (IPGui) biobank, where they were stored at −80 °C.

### 2.3. DNA Extraction

DNA was extracted from the tail or tissues (lungs and kidneys) of the micromammals using a DNeasy Blood and Tissue Kit (Qiagen, Courtaboeuf, France), following the manufacturer’s recommendations. Briefly, a small piece of tissue (0.4–0.6 cm in length for tails or 0.5 cm^2^ of tissue) was placed in 180 µL of Buffer ATL in a Fisherbrand 2 mL Pre-Filled Bead Mill Tube (containing 2.8 mm ceramic beads) and homogenized using a Fisher Scientific™ Bead Mill 24 Instrument (Fischer Scientific, Waltham, MA, USA). Then, 20 µL of proteinase K (providing by manufacturer) was added to the homogenate, which was then digested at 56 °C under slow shaking in a thermomixer until the samples were completely homogenized. Following quick centrifugation to remove debris, the obtained supernatants were used for DNA isolation using the kit protocol. The DNA was eluted in 100 uL of the AE elution buffer provided by the manufacturer. The negative control consisted of all the reagents without any tissues.

### 2.4. PCR Amplification

#### 2.4.1. Cytochrome b

In parallel to the morphological characterization in the field, we conducted genetical identification in the laboratory though PCR amplification of the cytochrome b gene (*Cytob*; size: 1140 bp) from DNA extracted from the tail samples using primers H15915 (5′-TCT CCA TTT CTG GTT TAC AAG AC-3′) and L14723 (5′-ACC AAT GAC ATG AAA AAT CAT CGT T-3′) [34,35]. The sizes of the amplification products were checked on an agarose gel (1%) and then sequenced using MinION Nanopore Technology at the IPGui by following the protocol available on the following website: https://nanoporetech.com/document/ligation-sequencing-amplicons-native-barcoding-v14-sqk-nbd114-24 (accessed on 15 July 2024). We found a perfect concordance between the physical and genetical identifications. All cytochrome results are available upon demand.

#### 2.4.2. *Lfb1* Amplification

Lung and kidney DNA extracts were tested for pathogenic *Leptospira* spp. using SYBR Green real-time PCR targeting part of the fibronectin-binding protein 1 (*Lfb1*) gene (331 bp) [36]. Each PCR was carried out in a 20 μL mixture containing 1 μL of the DNA template, 1 μL of each primer (0.4 mM) (primers: Lfb1-F: CATTCATGTTTCGAATCATTTCAAA and Lfb1-R: GGCCCAAGTTCCTTCTAAAAG), and 10 μL of the 2X Universal SYBR Green master mix (Biorad Life Science, Marnes-la-Coquette, France). An initial denaturation at 95 °C for 1 min was followed by 40 cycles of 10 s at 95 °C and 30 s at 60 °C. Finally, the samples were heated to 95 °C for 15 s, cooled down to 65 °C, and reheated from 65 °C to 90 °C at a rate of 0.1 °C per 5 s. A CFX96 real-time PCR detection system (Bio-Rad Laboratories, Hercules, CA, USA) was used for the amplification assays. Positive samples were included in each PCR run, using *Leptospira* DNA as the template, with *L. interrogans canicola* Hond Utrecht IV Canicola, *L. borgpetersenii* Sejroe M84 Sejroe, and *L. kirschneri* Grippotyphosa Moskva-V Grippotyphosa DNA in the positive controls for *L. interrogans*, *L. kirschneri*, and *L. borgpetersenii*, respectively (Table 1). These controls strains were obtained from the National Reference Center of Spirochetes (Paris, France), in charge of the *Leptospira* surveillance and located at Institut Pasteur in Paris.

A negative control (with PCR-grade water) was included in each run to rule out the possibility of cross-contamination. The reproducibility of the assays was assessed by repeating the DNA extraction and PCR assays at least twice.

### 2.5. Lfb1 Genotyping and Phylogeny Assignment

Samples that tested positive for the presence of *Leptospira* via PCR were analyzed to determine the species and species group using the *Lfb1* PCR described by Garcia-Lopez et al. [37]. *Lfb1* sequences are accessible in the *Leptospira* cgMLST database (https://bigsdb.pasteur.fr/leptospira/, accessed on 15 July 2024). All positive PCR products were subjected to Sanger sequencing (Eurofins Scientific, Colonia, Germany and Genoscreen, Lille, France). Sequence and phylogenetic tree analyses were performed using the software BioNumerics V7.6 (Applied-Maths, Saint-Martens-Latem, Belgium). The 12 *Lfb1* sequences used in the production of the phylogenetic tree are summarized in Garcia-Lopez et al. [37]. The *Lfb1* nucleotide sequences were deposited in GenBank under the accession numbers PQ876353 and PV185725 for lfb1-21-BA/MCT/GUINEA/2022 and lfb1-34-BA/MCT/GUINEA/2022, respectively.

### 2.6. Authorization to Carry Out Data Sampling

All necessary authorizations to perform the protocols in this study, particularly the micromammal collection in the villages, were obtained, including (1) an “accord de partages des advantages” (APA) signed by the Ministry of the Environment (Ministère de l’Environnement et du Développement Durable (MEDD), Office Guinéen des Parcs et Réserves de Faunes (OGPNRF)). The collection protocol is detailed in the “APA” document, which states that invasive samples were only collected from micromammals trapped in a house and thus considered “invasive animals”. (2) A scientific research license was specifically provided by the Fauna and Flora division of the MEDD to allow us to perform the scientific activities in villages close to protected forests (“forêt classée”). Moreover, the IPGui submitted and obtained a permit through the Nagoya protocol to potentially exchange data samples with regional, continental, or international partners while protecting the interests of Guinea. The team listed on the permit includes scientific and veterinary team members and the expert rangers of the local forest in question. Prior to any campaigns, the local rangers visited each village to explain the objectives of this study and how the activities would be performed (trapping in houses). The first day in each village was devoted to explaining the study to the community, including projecting a movie in the village (in the local language) to explain our activities in the field and the lab, an event that anyone in the village could attend.

## 3. Results

### 3.1. Results from Captured Mammals

A total of 42 animals were trapped in peridomestic (“marigot”) and domestic (houses or granaries) areas. Six micromammal species were identified: *Rattus* spp. (1/42, 2.38%), *Mus* spp. (5/42, 11.9%)*, Mastomys* spp. (12/42, 28.58%)*, Praomys* spp. (1/42, 2.38%)*, Lophuromys* spp. (10/42, 23.80%), and *Crocidura* spp. (13/42, 30.96%). The number and percentage of animals isolated in each village are detailed in Table 2.

### 3.2. Detection of Leptospira

**Figure 1:**
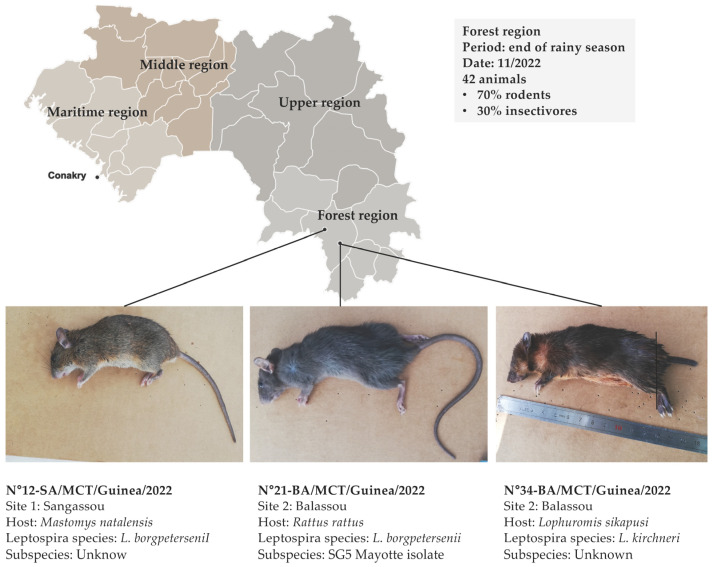
*Leptospira*-positive rodents captured during the field mission in November 2022 in the Guinean forest region. *L. borgpetersenii* was detected in an endemic commensal rodent (*Mastomys natalensis*) in Sangassou (12-SA) (8.615504, −9.475624) and an agricultural pest rodent (*Rattus rattus*) (21-BA) in Balassou (8.400137, −9.312699), both of which were trapped in houses, while *L. kirschneri* was detected in a wild rodent (*Lophuromys sikapusi*) (34-BA) living near the “marigot” (wetland) of Balassou. More information on the number of species captured is given in Table 2. MCT: Prefecture of Macenta.

Two *Leptospira* species were detected in the kidneys of three rodents, giving an overall prevalence of 7.1% (3/42) (Table 3): *L. borgpetersenii* was detected in one *Mastomys natalensis* in Sangassou (12-SA) and in a *Rattus rattus* in Balassou (21-BA), while *L. kirschneri* was also identified in a *Lophuromys sikapusi* in Balassou (34-BA) (Table 3 and Figure 1). These two *Leptospira* species are mostly specific to the African continent, as is *L. interrogans*, although we did not detect it in any of our samples [4,19].

### 3.3. Sequencing

**Figure 2:**
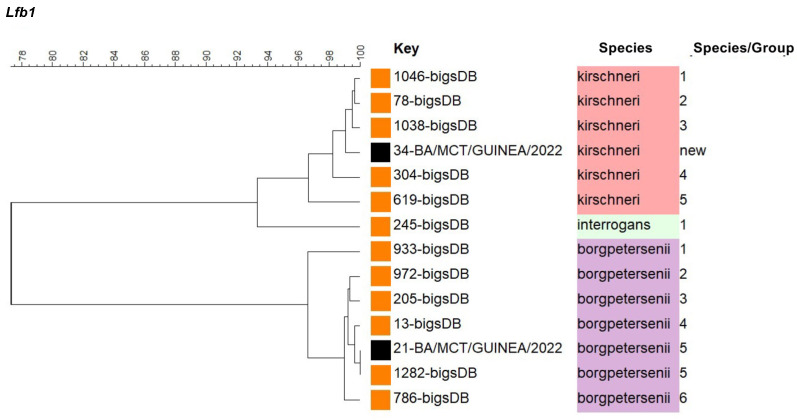
Phylogenetic tree inferred from *Leptospira* spp. detected in Guinean rodents based on partial sequence of the fibronectin-binding protein 1 (*Lfb1*) gene (331 bp). Orange boxes indicate reference strains from the BIGSdb, and black boxes indicate *Leptospira* spp. isolated from micromammals captured in Guinean forest villages: lfb1-34-BA/MCT/GUINEA/2022 (GenBank number: PV185725) and lfb1-21-BA/MCT/GUINEA/2022 (GenBank number: PQ876353). The BIGSdb accession numbers are indicated for the reference strains, and further information about the reference strains can be found in Garcia-Lopez et al. [37].

Phylogenetic analysis of the *Lfb1* gene allowed for more precise identification of the *Leptospira* species/subgroup (SG). The *R. rattus* (21-BA) carried *L. borgpetersenii* SG5, which is related to strains of the serogroup Pomona serovar Pomona (Figure 2). This profile was only found in human patients from the island of Mayotte, without the identification of the reservoir host. The *L. sikapusi* (34-BA) carried a new strain of *L. kirschneri* SG that did not match any sequence contained in the Bacterial Isolate Genome Sequence Database (BIGSdb). The *L. borgpetersenii* species infecting the *M. natalensis* (12-SA) could not be more precisely identified due to the poor quality of the sequence.

## 4. Discussion

Here, we provide new data on rodent-borne *Leptospira* in West Africa; despite having been characterized in many SSA countries, the epidemiological pattern of related diseases remains unclear [4,12,13]. There is a lack of data on leptospirosis in Guinea, and the data are limited to a study carried out in the capital, Conakry, in 2004 [26].

Considering the biodiversity of micromammals in the Guinean forest, we decided to study the southeast part of the country, especially villages close to the forest, in order to (1) give the population, which is often isolated, access to the available information on the risk of zoonoses; (2) fill the knowledge gap regarding *Leptospira* spp.; and (3) carry out micromammal trapping in peridomestic and domestic areas to identify the *Leptospira* spp. present in this area.

Compared to studies in urban areas, the sample in this study in terms of the number of animals captured was small, especially in the domestic area, with 42 isolated micromammals. Nevertheless, in a single 15-day campaign, three micromammals were found to be positive for *Leptospira*, showing, for the first time, its circulation in rural areas more than 800 km from Conakry. In addition, in the village Balassou, 22 micromammals were isolated over 3 days, 2 of which were positive (9%) for two different *Leptospira* species (confirmed via sequencing). A similar percentage was found (~9.1%) in 779 commensal micromammals that were sampled over 2 years in the center of Cotonou [19].

*Leptospira* were detected in an endemic commensal rodent (*M. natalensis*) and an agricultural pest rodent (*R. rattus*), both captured in houses, but also in a wild rodent (*L. sikapusi*) living near a wetland. The host potential in the Guinean forest is not limited to rodents and can include insectivores. A high prevalence was found in the native shrew *Crocidura olivieri* in two previous investigations in Benin [19,21]. As previously mentioned, shrews have been described as a reservoir for new hantavirus species [38]. In addition, the rodent species carried specific serogroups/serovars of *Leptospira* spp.: *L. borgpetersenii* was detected in *R. rattus*, and a new strain of *L. kirschneri* was detected in an *L. sikapusi* captured in the same village. This species and genotypic diversity within a limited area has been reported previously [19,20]. *L. borgpetersenii* and *L. kirschneri*, together with *L. interrogans*, are mostly found in SSA [4]. Compared to other rodents, *R. norvegicus* is globally associated with a very high prevalence of *L. interrogans* infection [39]. This high species-specific prevalence was found in Europe (in Lyon, France [40], and in Helsinki, Finland [41]), in South America (in Salvador, Brazil [42]), in Japan [43], and even in West African capitals such as Cotonou, Benin, with a prevalence of 45.2% [19]. This prevalence was more commonly found in African and EU urban cities compared to rural areas. No *R. norvegicus* were trapped in the rural villages that we visited, which may explain the lack of *L. interrogans* detected in our study area.

Trapping was performed once in each village and only provides a snapshot of the biodiversity and abundance of micromammals in villages in the Guinean forest. Our data showed that one *Leptospira* species infected both *Rattus rattus* and *Mastomys natalensis*, suggesting that many micromammals could be hosts for different stages of the *Leptospira* life cycle. Our results in terms of micromammal biodiversity and *Leptospira* prevalence cannot be extrapolated to the Guinean forest level as we concentrated our activities in a limited area outside a protected forest. In addition, it was difficult to distinguish whether it was the habitat or host-related determinants that affected the presence of pathogenic *Leptospira*. Extending the duration of the rural campaign would allow us to visit more villages and investigate other ecosystems to answer these questions.

We cannot rule out the possibility that some reservoirs may have been missed. Previous studies have demonstrated the high sensitivity of 16S rRNA sequencing, and the combination of two RT-PCR tests, including 16S rRNA, could increase the detection sensitivity for *Leptospira* spp. and might allow for the detection of a higher number of positive animals with Ct values above 30 [19]. Here, we studied a single gene, *Lfb1*; however, the use of only one target could skew detection towards the quantitatively dominant strain and result in a failure to efficiently detect mixed infections. Double and triple infections with *Leptospira* spp. have been described in Madagascar [44]. Techniques and target combinations to detect coinfections have been developed [19]. The 16S RNA/*Lfb1* combination will likely be favored in the future to efficiently detect mixed infections. To better understand host specificity for different *Leptospira* strains, how strains are transmitted between host species, and how these interactions vary depending on the landscape, PCR and sequencing approaches will likely be important tools. In this study, the lung/kidney samples were not kept in a culture medium to allow for *Leptospira* isolation in the lab; therefore, we were unable to detect any coinfection events.

## 5. Conclusions

Taken together, these results encourage us to carry out more missions. To study the biotic and abiotic factors that may influence the prevalence and genetic diversity of *Leptospira*, we need to (1) trap animals in different ecosystems in each natural region in Guinea; (2) extend the duration of the collection period to allow for the analysis of seasonal factors; and/or (3) repeat the campaign in each area in a longitudinal survey. Environmental samples, such as standing water samples, should also be included. The life cycle of *Leptospira* spp. takes place in free or soil-associated waters where they can infect vertebrate hosts [7,8]. *Leptospira* were detected at the end of the rainy season in November 2022. In other studies, these bacteria were detected at the beginning of the rainy season after low to moderate and moderate rainfall. While researchers believe that its prevalence in rodents is associated with rainfall [45], there is no substantial evidence to confirm an association between *Leptospira* circulation and rainfall. Several previous studies have suggested that the *Leptospira* infection risk also strongly depends on socio-environmental conditions [19,21].

Further studies will be carried out in the capital city of Conakry, from the center to the suburbs, to assess *Leptospira* circulation and identify the urban landscape elements that favor *Leptospira* circulation and the infection of multiple hosts. The origin of *Leptospira* on the African continent has not been clearly investigated. The phylogenetic proximity between *L. borgpetersenii* SG5 isolated from the *R. rattus* in Guinea in this study and that isolated from human cases in Mayotte suggests a potential role of maritime trade in *Leptospira* circulation in Africa, as has been reported for the Seoul virus through the introduction of rats from seaports [10]. In Conakry, a greater focus on seaport trade will allow us to identify the potential host/introduction route through combined phylogenetic and spatiotemporal analyses. For example, it has been suggested that, in Madagascar, a spillover of *L. mayottensis* from endemic rodents to the invasive black rat occurred [44].

## Figures and Tables

**Table 1:** Positive controls used for SYBR Green real-time PCR.

Species	Serogroup	Serovar	Reference Strain	Ct Values (nd, 1/1000) *		Melting T (°C)
*L. interrogans*	Canicola	Canicola	Hond Utrecht IV	15.63	27.88	81
*L. kirschneri*	Grippotyphosa	Grippotyphosa	Moskva V	16.32	28.78	82
*L. borgpetersenii*	Sejore	Sejroë	M84	22.16	31.73	83.5

* Positive control DNA dilution: 1/1000 or not diluted (nd).

**Table 2:** Micromammal species and abundance by village.

Site Name	Total	*Rattus* spp.	*Nanomys* spp.	*Mastomys* spp.	*Praomys* spp.	*Lophuromys* spp.	*Crocidura* spp.
Sangassou (SA)	17	3 (17.65)	3 (17.65)	2 (11.76)	0 (0)	1 (5.88)	8 (47.06)
Balassou (BA)	22	1 (4.6)	2 (9)	7 (31.8)	1 (4.6)	9 (41)	2 (9)
Ziama (ZI)	3	0 (0)	0 (0)	0 (0)	1 (33.3)	0 (0)	2 (66.7)
Total	42	4 (9.52)	5 (11.90)	9 (21.44)	2 (4.76)	10 (23.80)	12 (28.58)

(): % of species in village or in total (first row).

**Table 3:** Molecular detection of *Leptospira* species using *Lfb1* real-time SYBR Green PCR.

No.	Micromammal Species	Organ (DNA)	Ct Value	Melting T (°C)	Species and SG
12-SA	*Mastomys natalensis*	Kidney	32.32	83.5	*L. borgpetersenii* SG (na)
21-BA	*Rattus rattus*	Kidney	31.86	83.5	*L. borgpetersenii* SG5
34-BA	*Lophuromys sikapusi*	Kidney	24.97	82	*L. kirschneri* SG new

na: not assigned; SG: subgroup. The species and subgroup of sample No. 12 were not confirmed via sequencing.

## Data Availability

The original contributions presented in this study are included in the article. Further inquiries can be directed to the corresponding author.
